# Supplementary material for: Predictive Utility of Composite Child Feeding Indices (CCFIs) for Child Nutritional Status: Comparative Analyses for the Most Suitable Formula for Constructing an Optimum CCFI
Source: Int J Environ Res Public Health. 2022 May 29;19(11):6621. doi: 10.3390/ijerph19116621 (PMC9180453; doi:10.3390/ijerph19116621)
Supplement: Supplementary file 1 [file ijerph-19-06621-s001.zip › ijerph-1708593-SI.pdf]

Supplementary Materials

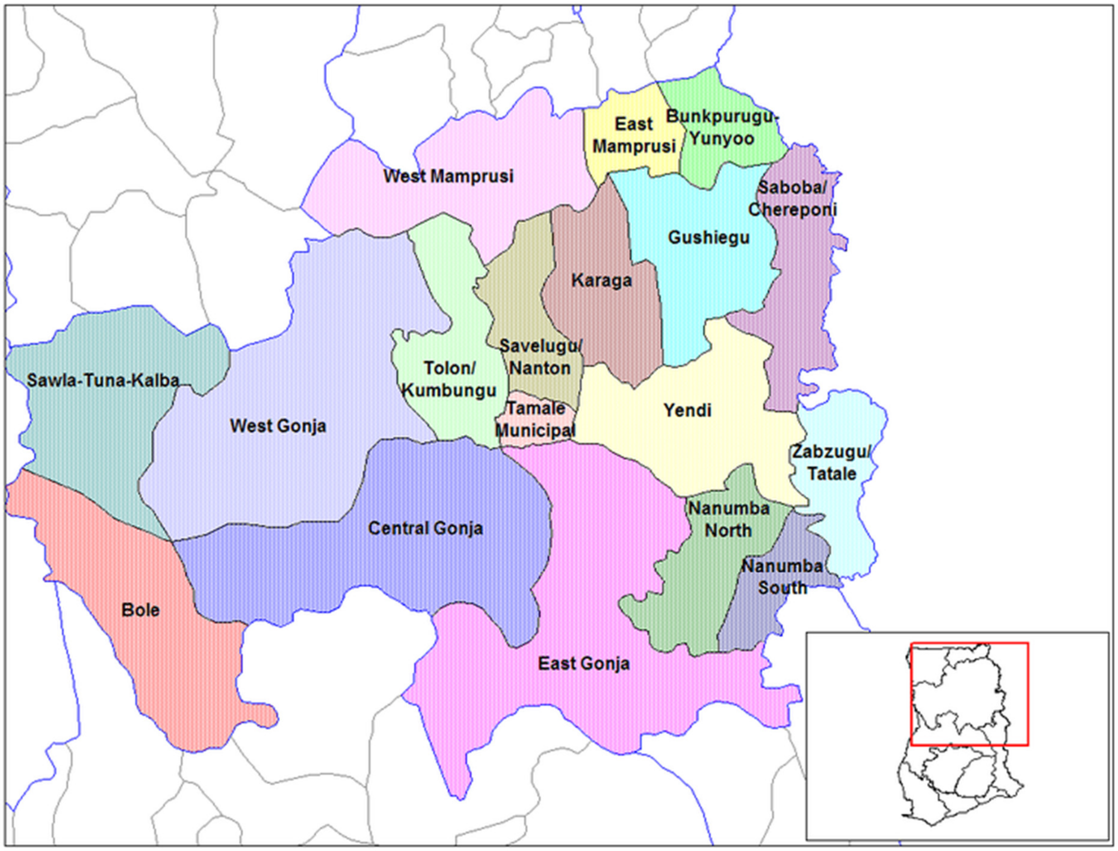

Figure S1. Illustrative Map of the Northern Region of Ghana (www.ghanaweb.)

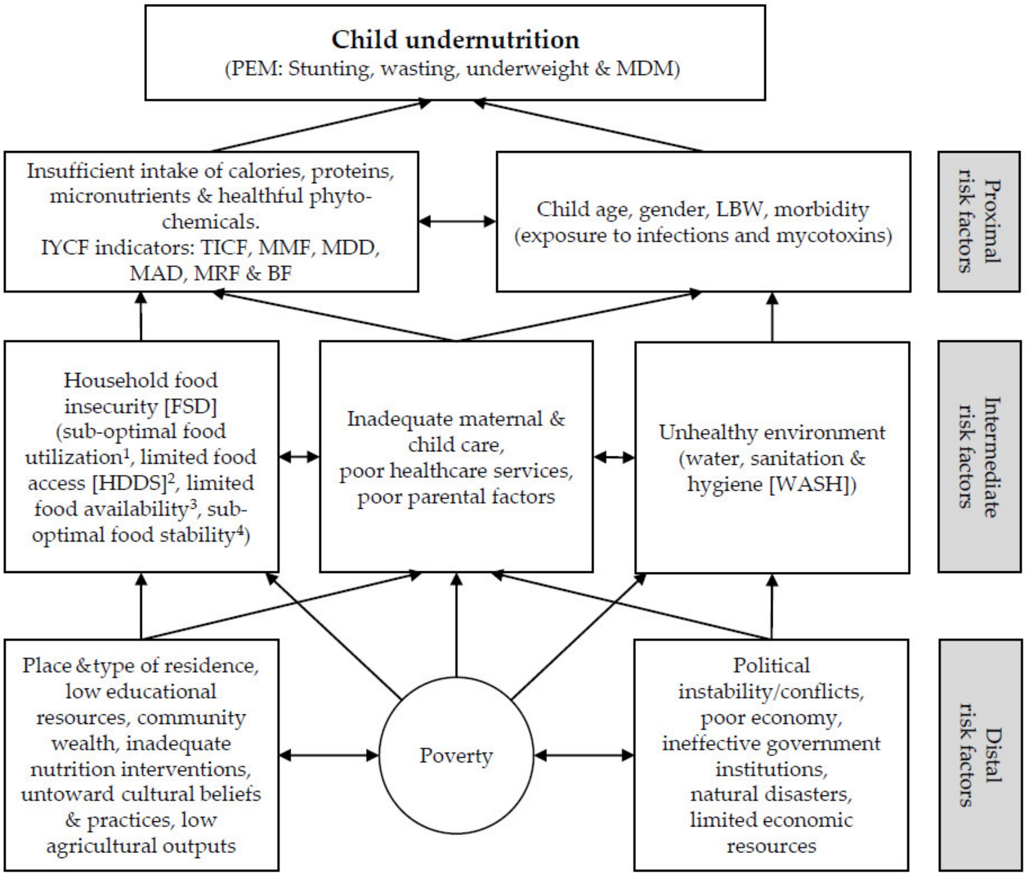

Figure S2. Conceptual Frame work for the Determinants of Malnutrition in Northern Ghana (Adopted and modified from Fanzo, 2012, Muller and Krawinkel, 2005 & UNICEF, 1998). [1] Sub-optimal food utilization: usage of nutritionally deleterious complementary food ingredient production practices such as Traditional Cereal Processing Methods (TCPMs), untoward Habitual Food Resource Utilization Practices (HFRUPs) such some traditional/indigenous food preparation methods, meal spoilage via lack of/poor storage facilities, post-harvest losses, poor knowledge of nutrition and untoward cultural beliefs and practices among other factors. [2] Limited food access (HDDS): low purchasing power, limited diversity of plant and animal food resources and inequitable household food /meal distribution [3] Limited food availability: inadequate food production, transportation and/or supply to the households' reach [4] Sub-optimal food stability: inconsistency in food production, supply, availability, access and utilization. FSD: Food Security Dimensions (Food Availability, Access, Utilization and Stability); HDDS: Household Dietary Diversity Score; IYCF: Infant and Young Child Feeding; LBW: Low Birth Weight; MAD: Minimum Acceptable Diet; MDD: Minimum Dietary Diversity; MDM: Micronutrient Deficiency Malnutrition; MMF: Minimum Meal Frequency; MRF: Micronutrient Rich Foods; BF: Breastfeeding; MRF: Micronutrient-Rich Foods; PEM: Protein Energy Malnutrition; TICF: Timely Introduction to Complementary Feeding.
